# Factors affecting length of hospital stay in chronic obstructive pulmonary disease patients in a tertiary hospital of Nepal: A retrospective cross-sectional study

**DOI:** 10.1016/j.amsu.2022.104246

**Published:** 2022-07-31

**Authors:** Pashupati Pokharel, Pratik Lamichhane, Pankaj Pant, Abhigan Babu Shrestha

**Affiliations:** a Maharajgunj Medical Campus, Institute of Medicine, Tribhuvan University, Kathmandu, Nepal; b Department of Pulmonology and Critical Care Medicine, Tribhuvan University Teaching Hospital, Kathmandu, Nepal; c M Abdur Rahim Medical College, Dinajpur, Bangladesh

**Keywords:** Chronic obstructive pulmonary disease, Clinical factors, Cross-sectional study, Length of stay, Nepal

## Abstract

**Background:**

Despite the increasing burden of chronic obstructive pulmonary disease in Nepal, studies analyzing the factors associated with inpatient length of hospital stay are lacking.

**Methods:**

This is a retrospective, cross-sectional hospital-based study conducted between July 2020 and July 2021 on patients admitted to the inpatient ward of Pulmonary and Critical Care Medicine of Tribhuvan University Teaching Hospital with a primary diagnosis of acute exacerbation of chronic obstructive pulmonary disease. The sample size of our study was 90. Clinical and demographic factors, blood investigation parameters, and treatment received were analyzed via univariate and multivariate analysis to find the factors associated with length of stay.

**Results:**

The mean age of chronic obstructive pulmonary disease patients was 68.84 ± 10.22 years, with 42.2% of males and 43.3% of current smokers. The length of hospital stay ranged from 2 to 25 days, with an average stay of 6.69 ± 4.02 days. Factors associated with length of stay are the number of comorbidities (p = 0.007), blood eosinophils at admission (p = 0.022), and use of mechanical ventilatory support (p < 0.001).

**Conclusions:**

Proper management of comorbidities and eosinophilic exacerbations as well as careful use of mechanical ventilatory support are required to further reduce the duration of hospital stay in chronic obstructive pulmonary disease patients.

## Highlights

• This study is the first of its kind identifying the factors associated with the length of hospital stay in COPD patients in Nepal.
• COPD is the fifth most common cause of inpatient admission in Nepal and per our study the length of stay was 6.69 days.
• The culprits identified for the increase length of stay were the number of comorbidities, blood eosinophils at admission and use of mechanical ventilator support.

## 1 Introduction

Chronic obstructive pulmonary disease (COPD) is a common preventable airway disease characterized by persistent airflow limitation [1]. Currently, with a 13.1% global prevalence, COPD is the third leading cause of death [2,3]. In Nepal, the national disease burden paradigm is shifting toward non-communicable diseases including COPD as the major cause of morbidity and mortality. Recent studies showed that the prevalence of COPD in Nepal is 11.7% with a higher prevalence among males, the elderly, and people with low educational status [4].

Hospitalization for acute exacerbation of COPD is recognized as a major event due to its negative effect on lung function, survival, risk of readmission, and quality of life [5,6]. The length of stay (LOS) in COPD patients is variable, ranging from 5 to 12 days [7,8]. Common factors associated with prolonged stay are older age, comorbidities, and socioeconomic deprivation. Moreover, a longer hospital stay is due to the vulnerability of patients requiring more attention from health personnel [9].

At present, COPD is the fifth most common cause for inpatient admission in Nepal. Despite the high national burden, studies analyzing potential factors associated with an inpatient stay in COPD patients are lacking. Therefore, this retrospective observational study was designed with the aim of finding the factors associated with the length of hospital stay in COPD patients in a tertiary teaching hospital of Nepal.

## 2 Materials and methods

This is a cross-sectional retrospective study conducted by reviewing the medical records of patients admitted with acute exacerbation of COPD (AECOPD) to the Tribhuvan University Teaching Hospital, Kathmandu, Nepal between July 2020 and July 2021. The research was conducted after ethical clearance from the Institutional Review Board of the Institute of Medicine, Tribhuvan University Teaching Hospital with reference number: 251/(6–11)E2/076/077. The procedures performed in this study were in accordance with the ethical standards of the institutional review board of the hospital and with the 1964 Helsinki Declaration and its later amendments or comparable ethical standards. This research has been registered in www.researchregistry.com, with unique identifying number (UIN): researchregistry7998. It is a retrospective study, thus formal consent from patients is not required.

For inclusion criteria, all patients discharged from Pulmonary and Critical Care Medicine wards with a primary diagnosis of AECOPD were included in the study. However, COPD patients leaving against medical advice (LAMA), patients discharged on their request (DOPR), inpatient death of COPD patients, and COPD patients admitted and discharged from the emergency department were excluded from the study.

The sample size of our study is calculated by formula S = Z^2^ P(1-P)/M^2^. Z is the standard normal variate, which is 1.96 for 95% confidence interval. P is the prevalence of COPD from the reference study, which is 11.7% [4]. M is the margin of error which we fixed at 7%. On putting these values, S = 81. Moreover, accounting for 10% data loss, 81 + 10% of 81 = 90. Therefore, the sample size for our study is 90.

First, the discharge files of the patients admitted with a primary diagnosis of AECOPD in the inpatient ward of Pulmonary and Critical Care Medicine were retrieved from the medical record section of the hospital. Then the individual parameters were recorded manually in the individual preconstructed datasheets. The datasheet contained variables based on demographic, clinical, blood investigation, and treatment parameters. The clinical and demographic parameters of interest were the age of COPD patients, gender, smoking status, number of comorbidities, degree of dyspnea, and presence of coexisting pneumonia. During data collection, the modified Medical Research Council (mMRC) guideline was used to grade the shortness of breath of COPD patients. The smoking status was classified into three types: current smokers, former smokers, and non-smokers as per the Centre for Disease Control and Prevention guidelines. The diagnosis of coexisting pneumonia was made on the basis of radiological findings during the hospital stay. Also, the number of comorbidities of each patient during admission was reported.

Similarly, the blood investigation parameters under consideration were arterial blood gas (ABG), blood hemoglobin (Hb), total leukocyte count (TLC), blood eosinophil level and serum creatinine (SCr). In ABG, the acidity of blood (pH), the partial pressure of carbon dioxide in arterial blood (PCO_2_) and bicarbonate ion concentration (HCO_3_^−^) were considered. As per the biochemistry laboratory of Tribhuvan University Teaching Hospital, the average values of these ABG parameters are: pH = 7.4, PCO_2_ = 40 mm of mercury, and HCO_3_^−^ = 24 milli equivalent per liter. Similarly, the reference range of other blood parameters are: Hemoglobin = 12–18 g per deciliter, Total Leukocyte Count = 4000–11000 cells per milliliter, Eosinophils = 1–6% of TLC, Creatinine = 60–115 μmol per liter.

Regarding treatment, the use of inhalation medications, systemic steroids, antibiotics, and mechanical ventilation (non-invasive or invasive) was recorded. However, the number of medications and their individual doses under these categories were not reported. The length of stay was calculated based on the date of admission and the date of discharge. However, the discharge destination and the cost factors associated with the length of stay were not taken into consideration in the study.

For statistical analysis, the parameters from the datasheet were first entered into an Excel spreadsheet (Microsoft Corp., 2019). All analyses were done in Statistical Package for the Social Sciences (SPSS), version 26.0 (IBM Corp., Armonk, N.Y., USA). Categorical variables were expressed in frequency and percentage, whereas numerical variables were expressed in terms of mean, median, and standard deviation. The length of stay was non-normal (high skewness), so nonparametric tests such as the Mann Whitney *U* test, Wilcoxon W test, and Kruskal Wallis H test were utilized to find the potential factors associated with the length of stay in COPD patients. During univariate analysis, any parameter with a p-value of <0.05 was considered to have a significant association with length of stay. Finally, all significant factors were entered into the final multivariate linear regression model as independent variables to find the final association.

## 3 Results

### 3.1 Patient characteristics

Among the 90 COPD patients, the mean age was 68.84 ± 10.22 years with 42.2% being males. Of them, 43.3% were current smokers, 14.4% did not have any comorbidity, 43.3% had mMRC grade IV dyspnea at presentation, and 44.4% had coexisting pneumonia. The details of clinical and demographic characteristics are shown in Table 1. During admission, more than 80% of patients had respiratory acidosis, 68.9% had normal hemoglobin, 67.8% had normal total leukocyte count, 53.3% had normal eosinophil count, and 60% had normal serum creatinine. The details of blood investigation parameters are shown in Table 2.

**Table 1:** Clinical and demographic profile of COPD patients.

Parameter	Sub Parameter	Value
Age range	50–62 years	26 (28.90%)
	62–70 years	26 (28.90%)
	70–77 years	22 (24.4%)
	77–90 years	16 (17.8%)
Gender	Male	38 (42.2%)
	Female	52 (57.8%)
Smoking status	Current smoker	39 (43.3%)
	Former smoker	35 (38.9%)
	Nonsmoker	16 (17.8%)
Number of comorbidities	0	13 (14.4%)
	I	31 (34.4%)
	II	33 (36.7%)
	III	8 (8.9%)
	IV	4 (4.4%)
	V	1 (1.1%)
Dyspnea	Grade III	51 (56.7%)
	Grade IV	39 (43.3%)
Coexisting pneumonia	Yes	40 (44.4%)
	No	50 (55.6%)

**Table 2:** Blood investigation parameters of COPD patients.

Parameter	Sub Parameter		Value
Arterial Blood Gas (ABG)	pH	<7.4	75 (83.3%)
		>7.4	15 (16.7%)
	PCO_2_	>40 mmHg	76 (84.4%)
		<40 mmHg	14 (15.6%)
	HCO_3_^−^	>24 mmol/l	69 (76.7%)
		<24 mmol/l	21 (23.3%)
Hemoglobin (Hb) (g/dl)	Normal Hb (12–18)		62 (68.9%)
	Low Hb (<12)		21 (23.3%)
	High Hb (>18 g/dl)		7 (7.8%)
Total Leukocytes Count (cells/μl)	Normal count (4000–11000)		61 (67.8%)
	High count (>11000)		28 (31.1%)
	Low count (<4000)		1 (1.1%)
Eosinophils (% of TLC)	Normal count (1–6%)		48 (53.3%)
	Elevated count (>6%)		6 (6.7%)
	Low count (<1%)		36 (40%)
Absolute eosinophil count (cells/μl)	<300		58 (65%)
	>300		32 (35%)
Serum Creatinine (SCr) (mmol/l)	Normal SCr (60–115)		54 (60%)
	Elevated SCr (>115)		17 (18.9%)
	Low SCr (<60)		19 (21.1%)

During the course of their hospital stay, 97.7% of patients received inhaled medication in any form, all received systemic antibiotics, 72.2% received systemic steroids, and 23.3% required invasive ventilation. The average length of hospital stay was 6.69 ± 4.02 days (median LOS = 6 days, standard deviation = 4.02) ranging from 2 to 25 days. The details of treatment parameters and length of stay are shown in Table 3.

**Table 3:** Treatment parameters and length of stay of COPD patients.

Treatment Parameters			
Inhaled medications	Yes	87 (96.7%)	
	No	3 (3.3%)	
Systemic antibiotics	Yes	100%	
	No	–	
Systemic steroid	Yes	65 (72.2%)	
	No	25 (27.8%)	
Mechanical ventilation	Noninvasive ventilation	69 (76.7%)	
	Invasive ventilation	21(23.3%)	
**Length of Stay**			
Range	2–4 days		28 (31.1%)
	4–6 days		29 (32.2%)
	6–8 days		15 (16.7%)
	8–25 days		18 (20.0%)

### 3.2 Factors associated with length of stay

As the length of stay was a non-normal dependent variable (Skewness = 2.034, Kurtosis = 5.158), nonparametric statistical tests were utilized to find the factors associated with LOS. In univariate analysis, the number of comorbidities (p = 0.01), dyspnea grade at presentation (p < 0.001), eosinophil percentage (p = 0.017), use of inhalational medications (p = 0.007) and the use of mechanical ventilation (p < 0.001) had significant association with LOS. However, after the final multivariate analysis, the number of comorbidities (p = 0.007), the percentage of eosinophils (p = 0.022) and use of mechanical ventilation (p < 0.001) were significantly associated with LOS in COPD patients. The details are shown in Table 4.

**Table 4:** Parameters with significant association with length of stay.

Parameter	Univariate analysis	Multivariate regression analysis	
	P value	P value	B with 95% Confidence interval
Gender	0.057	–	–
Age	0.923	–	–
Smoking	0.223	–	–
Number of comorbidities	**0.01**	**0.007**	0.688 (0.064–1.313)
Dyspnea grade	**< 0.001**	0.607	0.409 (−0.917 to 1.734)
Pneumonia	0.110	–	–
Eosinophil percentage	**0.017**	**0.022**	−0.585 (−1.126 to −0.043)
Absolute eosinophil count	**0.008**	0.182	0.004 (−0.001 to 0.009)
Total leukocyte count	0.194	–	–
Hemoglobin	0.147	–	–
Serum creatinine	0.202	–	–
pH	0.541	–	–
pCO_2_	0.805	–	–
HCO_3_^−^	0.400	–	–
Inhalational medications	**0.007**	0.249	−2.078 (−5.296 to 1.140)
Systemic steroid	0.653	–	–
Mechanical ventilation	**< 0.001**	**< 0.001**	−5.304 (−7.006 to −3.601)

## 4 Discussion

This cross-sectional study was conducted with the aim of identifying the factors associated with the length of hospital stay in COPD patients in a tertiary teaching hospital of Nepal. To the best of our knowledge, this study is the first of its kind in Nepal to date. The patient's demographic profile, clinical profile, blood test results at admission, and length of stay were analyzed separately to find the factors associated with an inpatient stay in COPD patients. Our study found that the number of comorbidities, eosinophils count, and mechanical ventilatory support at admission was significantly associated with the length of stay in COPD patients. However, length of stay in our study was not associated with age, gender, smoking status, coexisting pneumonia, use of systemic steroids, ABG parameters at admission, hemoglobin level, leukocyte count and serum creatinine.

The average inpatient length of stay in COPD patients in our study was 6.69 days. Studies from different countries have shown considerable heterogeneity and geographical variation in the length of stay in COPD patients. The length of hospital stay in COPD patients is variable throughout the world, ranging from 11.64 days in the USA, 6–7 days in European countries, 7.8 days in Australia, 5 days in Hongkong, 9.38 days in China and 12.28 days in Macao [7,8,[10], [11], [12], [13]]. Therefore, on comparing these, the LOS of COPD patients is similar to that in European countries and Australia. However, the LOS is lower compared to China, USA and Macau. This variation is probably due to the variable mean age of COPD patients and the diverse health systems in the countries.

Our study showed a significant association between length of stay with the number of comorbidities COPD patients were having at admission. Four out of five COPD inpatients in this study had at least one comorbidity, which was similar to previous studies [8,14,15]. A similar association has been established by various studies, thus reaffirming comorbidities as an important predictor of prolonged hospitalization [10,[16], [17], [18], [19]]. This association is due to compromised lung function leading to increased risk for other diseases or complications [[20], [21], [22]].

Length of stay was significantly associated with the blood eosinophil level in our study. The role of eosinophils in COPD exacerbation and LOS is quite controversial. The prolonged stay might be associated with a greater degree of inflammation and airway obstruction in eosinophilic exacerbation [23]. The study by Simon Couillard et al. showed that blood eosinophilia was associated with a shorter time to first COPD-related readmission and a greater number of 12 months exacerbations [24]. Contrary to this, study of Agrusa et al. showed that in severe acute exacerbations of COPD requiring hospitalization, blood eosinophilia was associated with a prompt response to treatment with a shorter hospital stay [25]. Similarly, the study of Greulich et al. showed that patients with low eosinophils had a longer median time in the hospital compared to patients with high eosinophils [26]. Therefore, further prospective studies are required to analyze the relation between blood eosinophilia and COPD exacerbation.

Moreover, the length of stay was significantly associated with the use of mechanical ventilatory support for the COPD patients during the inpatient stay. Similarly, an association between inpatient stay and mechanical ventilation has been seen in studies from India and Macau [8,27]. The study of Lindenauer et al. showed that COPD patients treated with noninvasive ventilation at the time of hospitalization had lower inpatient mortality, shorter length of stay, and lower costs compared with those treated with IMV [28]. The longer stay in patients with invasive ventilation is probably due to the critical nature of the exacerbation of COPD.

Despite the novelty, our study has certain limitations. As the study is of retrospective nature, certain variables were limited during the analysis due to the unavailability of the electronic database system in the hospital. Therefore, some of the potentially important predictors might have been missed. The cost factor associated with hospitalization was not studied, which might have a potential effect on the length of stay. Also, as the sample size of the study is small and is conducted in a single, the findings of this study need to be cautiously interpreted by the clinicians.

## 5 Conclusions

A higher number of comorbidities, high eosinophil count, and use of mechanical ventilation was associated with a prolonged hospital stay in COPD patients in Nepal. Proper management of comorbidities in COPD patients and careful use of mechanical ventilatory support are required to further reduce hospital stay duration. Nevertheless, further large prospective studies on COPD are required to speculate on other factors associated with a longer hospital stay, morbidity, and mortality in Nepal. As the burden of COPD is ever increasing in a lower-middle-income country like Nepal, preventive measures such as smoking cessation, protection from domestic and occupational smoke, and dust, along with the adoption of healthy health habits are of utmost importance.

## Ethical approval

Ethical approval taken from the Institutional Review Board of the Institute of Medicine, Tribhuvan University Teaching Hospital reference number:

251/(6–11)E2/076/077.

## Sources of funding

This research was supported by Undergraduate Health Research Grant from Nepal Health Research Council (Ref no. 1583). The funders had no role in the design and conduct of the study, in the collection, management, analysis, and interpretation of the data.

## Author contributions

Pashupati Pokharel: Research idea, study design, data acquisition, data analysis and interpretation, statistical analysis, manuscript drafting.

Pratik Lamichhane: Data acquisition, statistical analysis, manuscript drafting.

Pankaj Pant: Manuscript drafting, supervision/mentorship.

Abhigan Babu Shrestha: manuscript drafting.

## Registration of research studies

1. Name of the registry: researchregistry.com
2. Unique Identifying number or registration ID: researchregistry7998
3. Hyperlink to your specific registration (must be publicly accessible and will be checked): https://www.researchregistry.com/browse-the-registry#home/registrationdetails/62a5bba2774003001e2c9453/

## Guarantor

Pashupati Pokharel, Abhigan Babu Shrestha.

## Consent

The study was retrospective cross-sectional which didn't required consent of the patient, database were seen.

## Data availability

The data supporting the results will be made available on a reasonable request to the corresponding author.

## Declaration of competing interest

Pashupati Pokharel received funding from the Nepal Health Research Council.
